# Impact of a Multifaceted Pharmacist-Led Intervention on Antimicrobial Stewardship in a Gastroenterology Ward: A Segmented Regression Analysis

**DOI:** 10.3389/fphar.2020.00442

**Published:** 2020-04-15

**Authors:** Yaling Du, Jing Li, Xinchun Wang, Xi Peng, Xiaoyi Wang, Wenying He, Yan Li, Xuemei Wang, Qiuxia Yang, Xinping Zhang

**Affiliations:** ^1^ School of Medicine and Health Management, Tongji Medical College, Huazhong University of Science and Technology, Wuhan, China; ^2^ First Affiliated Hospital, School of Medicine, Shihezi University, Shihezi, China

**Keywords:** impact assessment, pharmacist role, multifaceted intervention, antimicrobial stewardship, gastroenterology, segmented regression

## Abstract

**Background:**

Irrational use of antimicrobial agents for gastrointestinal diseases deserves attention, but corresponding antimicrobial stewardship programs (ASPs) are generally not a priority for managers. We conducted this study to evaluate the effectiveness of multifaceted pharmacist-led (MPL) interventions in the gastroenterology ward (GW) to provide evidence for the efficacy of ASPs in a non-priority department.

**Methods:**

This was an interventional, retrospective study implemented in China. The MPL intervention lasting 1.5 years involved daily ward rounds with physicians, regular review of medical orders, monthly indicator feedback, frequent physician training, and necessary patient education. Data on all hospitalized adults receiving antibiotics was extracted from the hospital information system over a 36-month period from January 2016 to December 2018. Segmented regression analysis of interrupted time series was performed to evaluate the effect of the MPL interventions (started in July 2017) on antibiotic use and length of hospital stay, which was calculated monthly as analytical units.

**Results:**

A total of 1763 patients receiving antibiotics were enrolled. Segmented regression models showed descending trends from the baseline in the intensity of antibiotic consumption (coefficient = −0.88, *p* = 0.01), including a significant decline in the level of change of the proportion of patients receiving combined antibiotics (coefficient = −9.91, *p* = 0.03) and average length of hospital stay (coefficient = −1.79, *p* = 0.00), after MPL interventions. The MPL interventions led to a temporary increase in the proportion of patients receiving antibiotics (coefficient = 4.95, *p* = 0.038), but this was part of a declining secular trend (coefficient = −0.45, *p* = 0.05).

**Conclusion:**

The MPL interventions led a statistically significant decline in the number of patients receiving antibiotics, the antibiotic consumption, and the average hospital stay post-intervention compared to the pre-intervention phase of the study. Health policymakers should actively practice MPL interventions by clinical pharmacists in ASPs in those departments that are not included in priority management.

## Introduction

In recent years, the effectiveness of antimicrobial drugs for treating common infections has rapidly decreased, and widespread antimicrobial resistance (AMR) has become an ongoing threat to public health (Prestinaci et al., 2015; Ferri et al., 2017). The World Health Organization (WHO) adopted “antimicrobial resistance” as the theme for World Health Day 2011 and introduced a policy package for governments to take critical action against antimicrobial resistance (Leung et al., 2011). Many countries implemented antimicrobial stewardship programs (ASPs) to combat bacterial resistance (Howard et al., 2015). ASPs are a coherent set of strategies that can promote the appropriate use of antibiotics (Dellit et al., 2007; Davey et al., 2017; Dyar et al., 2017). The ASP policy of China was implemented in 2012, and the guidelines for clinical application of antimicrobials were updated in 2015 (Qu et al., 2019).

Although the worldwide development and implementation of ASPs differs on a regional level (Howard et al., 2015; Bishop, 2016), the consensus was that ASP teams should be multidisciplinary, with pharmacists as the core members that can optimize the utilization of antimicrobials (Brink et al., 2017; Wang et al., 2018). Clinical pharmacists are professionals who provide patients with comprehensive drug management and related care in all medical areas. They are licensed pharmacists with specialized advanced training, who play an important role in promoting the optimal use of antimicrobials, reducing the transmission of infections, and educating health professionals, patients, and the public (ASHP statement, 2010; American College of Clinical, 2014). The effect of pharmacists’ actions in ASPs was well documented in the United States (Barlam et al., 2016) and the United Kingdom (National Institute for Health and Care Excellence Guideline, 2015), where pharmacists were always involved in ASPs (Chou et al., 2016). Since the establishment of the system of clinical pharmacists in China in 2002, clinical pharmacists have been increasingly involved in ASPs in hospitals (Xiao and Li, 2013; Li et al., 2017; Zhang et al., 2017). Pharmacist-led interventions have yielded excellent results in many areas (Kooij et al., 2016; van Eikenhorst et al., 2017; Ravn-Nielsen et al., 2018; van der Laan et al., 2018). However, the studies that have been performed on ASPs for hospital inpatients led by pharmacists have received insufficient attention in developing countries (Davey et al., 2017; Sakeena et al., 2018).

Previous studies have shown that multifaceted pharmacist-led interventions can lead to more optimized use of medication, as well as reduced number of hospital visits and length of stay (Ravn-Nielsen et al., 2018; Skjot-Arkil et al., 2018). Moreover, pharmacists-driven antimicrobial stewardship interventions can effectively improve guideline compliance, timely administration of antibiotics, sustainable patient outcomes, and reduce the mean costs of antimicrobial therapy (Dunn et al., 2011; Messina et al., 2015; Brink et al., 2017; Wang et al., 2018). Studies on intervention in bacteremia (Wenzler et al., 2017), urine culture (Almulhim et al., 2019), perioperative prevention of infection (Sun, 2013), and surgical infection (Brink et al., 2017; Mahmoudi et al., 2019) were conducted in emergency departments, respiratory departments, surgical departments, and intensive care units. However, it should not be overlooked that bacterial resistance due to irrational use of antibiotics in gastroenterology is also a challenge (Fernandez et al., 2016; Piano et al., 2019). Bedini et al. performed a multidisciplinary team intervention in an ASP in an Italian gastroenterology ward (GW), which had a positive impact on reducing the consumption of antibiotics (Bedini et al., 2016). However, very little is known about multifaceted pharmacist-led (MPL) interventions in ASPs in the GW.

In this study, we used segmented regression analysis of interrupted time series (ITS) to assess the effect of MPL interventions in ASPs in a GW. The segmented regression analysis of ITS is a powerful quasi-experimental approach in which data are summarized at spaced intervals before and after the intervention (Bernal et al., 2017). It can be used to evaluate changes in the levels and trends of the outcome after the intervention while controlling for pre-existing trends and temporal confounders (Wagner et al., 2002). Therefore, the intent of this quasi-experimental study was to explore whether the interventions of MPL reduced the use of antibiotics, to determine the effectiveness of MPL interventions in a GW, and to provide a scientific basis for detailed evidence-based management of ASPs in non-priority departments.

## Materials and Methods

### Study Setting and Patients

This study was performed at the GW of a 1500-bed academic teaching hospital in Xinjiang, China. The GW had 62 beds and 8305 discharged patients during the study period (January 1, 2016, to December 31, 2018). Patients who had used at least one antibiotic were selected as subjects, the principal diagnosis and antimicrobial utilization data of these patients were extracted from the hospital information system, and the patients’ personal details (the patient’s name, phone number, id number, home address) were hidden. The data collected from January 1. 2016 to December 31. 2018, 18 months prior to the intervention (January 1. 2016 to June 30. 2017) and 18 months after the start of the intervention (July 1. 2017 to December 31. 2018), included demographic information, principal diagnosis, specific information about antimicrobial use (name, dose, duration of use, category, etc.).

### Multifaceted Pharmacist-Led Interventions

In July 2017, a clinical pharmacist with a professional qualification in gastroenterology was assigned to the GW to provide multifaceted interventions for rational use of antibiotics. The interventions were conducted according to the updated Chinese Guidelines for the Clinical Application of Antimicrobial Agents (National Health Commission of the People’s Republic of China, 2015a). The multifaceted interventions included (1) daily ward round: made ward rounds with the physician every day (working days only) to assess the patients’ diagnosis, medication, and laboratory sensitivity results, and gave advice to the physician to determine the optimal drug treatment; (2) regular review of medical orders: checked each patient’s temporary and long-term medical orders, and gave feedback and explanation of the problematic orders to the physician; (3) monthly indicator feedback: summarized and gave feedback on the department’s antimicrobial management indicators related to performance appraisal at the end of each month; (4) frequent physician training: provided physicians with training on the rational use of antimicrobial drugs, including gathering physicians together for training and assessment, and daily communication; and (5), necessary patient counselling and education: gave one-on-one medication guidance and education for patients in need (e.g., older patients, patients receiving at least 5 medications, or patients after endoscopic surgery).

### Outcomes

We reported four indicators to assess the antimicrobial use and clinical outcomes.

The intensity of antibiotic consumption (IAC) was defined as the cumulative number of defined daily doses (DDDs) of antibiotics in hospitalized patients per 100 patient days in the same period (DDDs/100pd). According to the WHO Collaborative Centre for Drug Statistics Methodology (WHO, 2003), the DDDs equivalence was defined as the usage amount multiplied the pack size divided by the defined daily dose (DDD), a measurement developed by the WHO to compare drug consumption, DDDs = (usage amount × pack size/DDD) (Tang et al., 2018).

The proportion of receiving antibiotics (PRA) = Number of hospitalizations receiving antibiotics/Number of hospitalizations over the same period × 100%.

The proportion of receiving combined antibiotics (PRCA) = Number of hospitalizations receiving more than one antibiotics/Number of patients that received antibiotics over the same period × 100%.

The average length of hospital stay (ALoS) = Total hospitalization days of patients who received antibiotics/Number of patients who received antibiotics in the same period.

### Statistical Analysis

The outcomes were assessed by segmented regression analysis of ITS data (Wagner et al., 2002; Bernal et al., 2017), calculated monthly as analytical units in this study.

The segmented regression model (the initial segmented linear regression model) for each indicator was described as follows:

$$Y=\beta_{0}+\beta_{1}*\mathrm{time}+\beta_{2}*\mathrm{intervention}+\beta_{3}*\mathrm{time}*\mathrm{intervention}+\epsilon$$

where Y represents the IAC, PRA, PRCA, or ALoS of the GW from January 1. 2016 to December 31. 2018. Time represents the value of the time-variable in months, 1 = January 2016, 2 = February 2016, 3 = March 2016 … Intervention is a binary variable indicating time periods before and after intervention. The coefficient β_0_ estimates the baseline level of the outcomes in January 2016 (baseline level); β_1_ estimates the change that occurs with each month before MPL interventions (baseline trend/slope); β_2_ and β_3_ represent changes in the levels and trends of indicators during the intervention (compared with baseline level and trend), which indicate transient and long-term effects of intervention, respectively. The term ϵ is a residual error. Residual analysis was used to assess the presence of serial autocorrelation. Autocorrelation was assessed by computing the Durbin-Watson statistic. The generalized least-squares method was used to adjust the model and re-evaluate the effects of intervention when a statistically significant autocorrelation was detected. Categorical variables were assessed using the Chi-squared test or Fisher’s exact test; continuous variables were assessed using the t-test. All statistical analyses were conducted using STATA version 12.0 (STATA Corp, College Station, TX, USA), and differences with *P* < 0.05 were considered statistically significant.

## Results

### Patient Characteristics

**Table 1** presents the characteristics of the patients who received at least one antibiotic during the time that they were admitted to the GW. Before and after the intervention period, 883 and 880 patients received antimicrobial therapy, respectively. The mean age of the patients in the periods before and after intervention was 61.97 years and 62.17 years, respectively. About 55% of these patients were male. The top 5 principal diagnoses were acute pancreatitis, choledocholithiasis, choledocholithiasis with cholangitis, cirrhosis, and chronic gastritis (*Helicobacter pylori*). There were no significant differences in age, gender, and the number of top 5 diagnoses of the admitted patients.

**Table 1:** Patient’s characteristics for receiving antibiotics during the pre- and post-intervention periods.

Characteristic	Pre-intervention	Post-intervention	*p*-value
Admissions, n	883	880	
Age, mean (SD)	61.97 (15.75)	62.17 (16.87)	0.79^a^
Male	482 (54.59)	484 (55.0)	0.86
Principal diagnosis (top 5), n (%^b^)			0.19
Acute pancreatitis	140 (15.86)	160 (18.18)	0.51
Choledocholithiasis	135 (15.29)	124 (14.09)	
Choledocholithiasis with cholangitis	59 (6.68)	73 (8.29)	
Cirrhosis	74 (8.38)	68 (7.72)	
Chronic gastritis (Helicobacter pylori)	43 (4.87)	42 (4.77)	

Data are number (%) unless otherwise indicated. All p-values calculated using Chi-square unless otherwise noted. SD, standard deviation; ^a^t-test; ^b^the percentage of the patients with the indicated diagnosis who received antibiotics as a percentage of the total patients in the same period.

### Time Series of Antimicrobial Use and Length of Hospital Stay in the Period of 2016–2018

**Figure 1:**
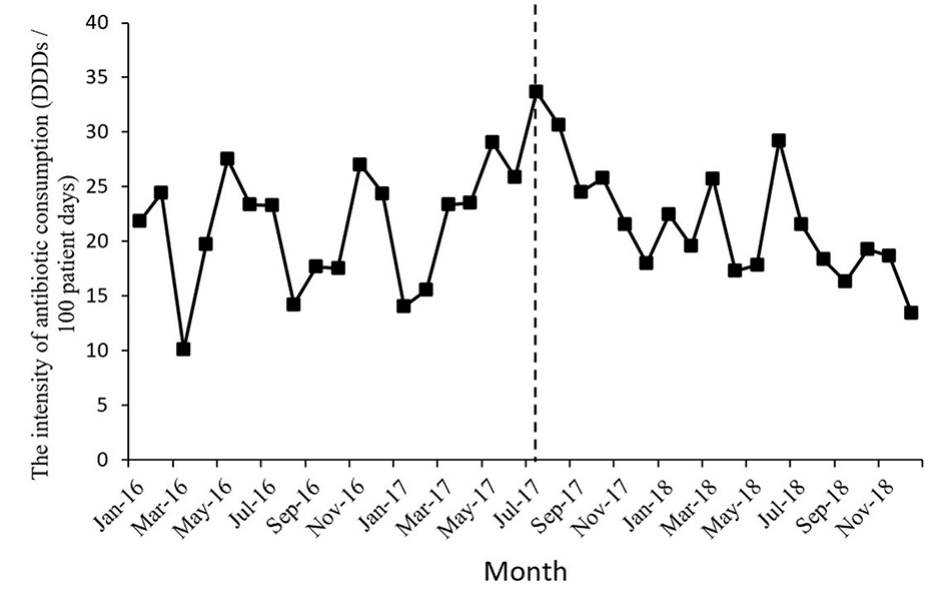
The change of the intensity of antibiotic consumption per month pre- and post-intervention for the gastroenterology ward. The vertical line separated two segments (before and after the MPL intervention).

**Figure 2:**
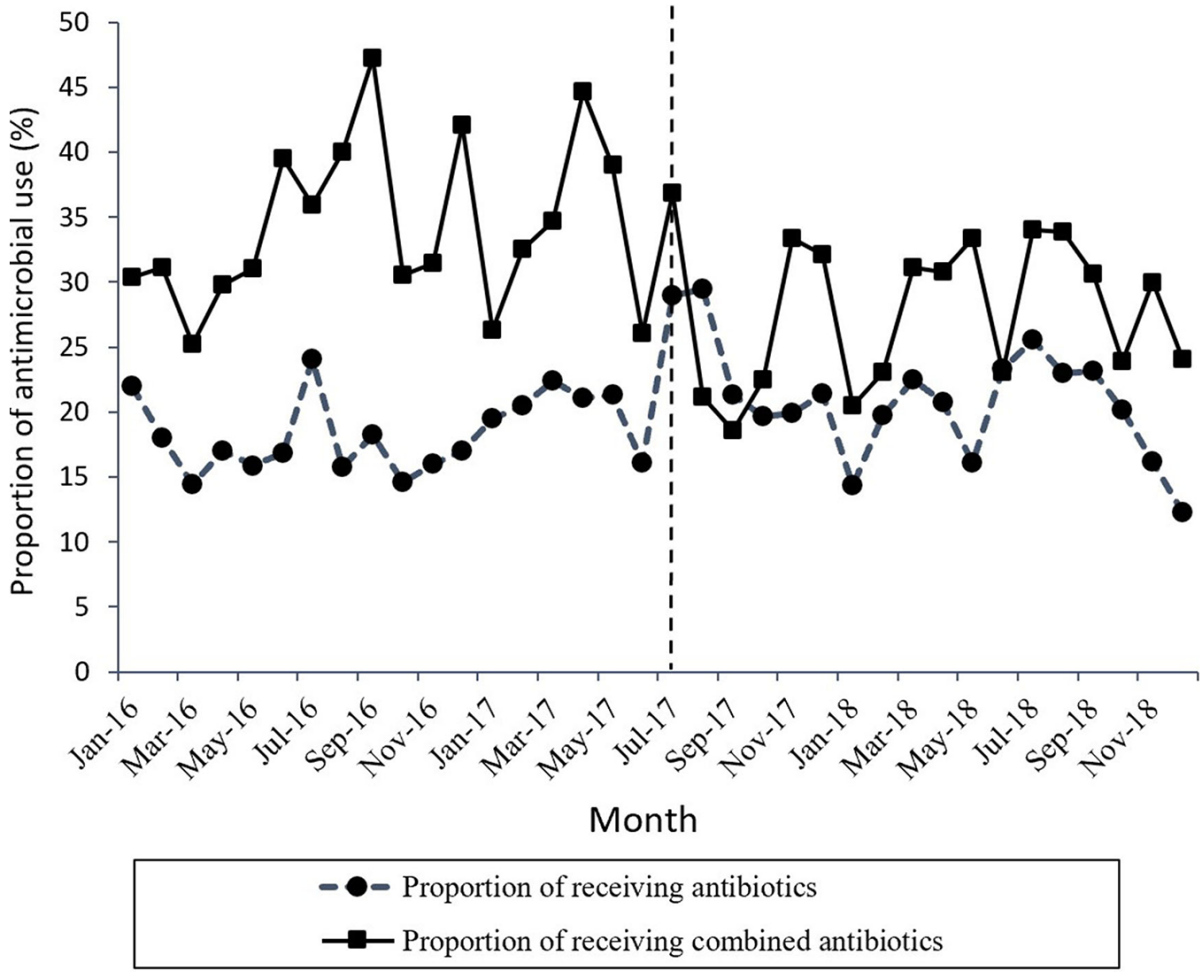
The change in the proportion of receiving antibiotics and the proportion of receiving combined antibiotics per month pre- and post-intervention. The vertical line separated two segments (before and after the MPL intervention).

**Figure 3:**
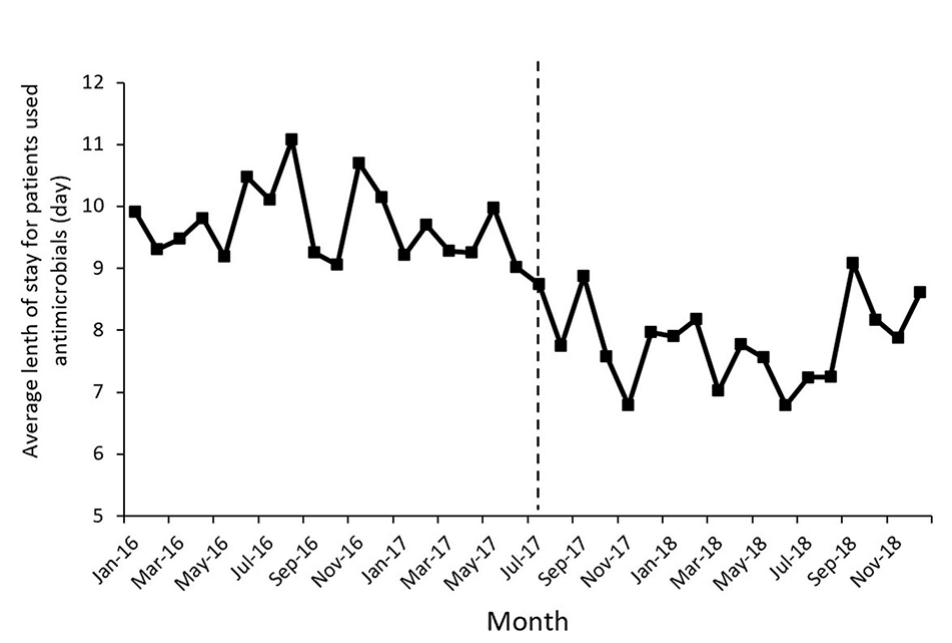
The change of the average length of stay for patients used antimicrobial per month pre- and post-intervention. The vertical line separated two segments (before and after the MPL intervention).

**Figure 1** shows the time series of monthly values for IAC. **Figure 2** presents the time series of monthly values for PRA and PRCA. **Figure 3** displays the time series of monthly values for ALoS.

### Results of the Segmented Regression Analysis

**Table 2** lists the detailed results of the levels and trend changes before and after the MPL intervention. **Figures 1**–**3** show that antibiotics use or length of hospital stay peaked during certain seasons, so the regression model included seasonality to control for confounding. Furthermore, we examined the presence of serial autocorrelations by residual analysis. There were no auto-correlative effects in the four models.

**Table 2:** Results of the segmented regression analysis of IAC, PRA, PRCA, and ALoS.

Outcomes	Coefficient	Standard error	*t* - statistic	*P* - value
IAC (DDDs/100pd) (DW = 1.97)				
Level before MPL interventions (β_0_)	17.36	2.53	6.86	0.00
Trend before MPL interventions (β_1_)	0.35	0.18	2.02	0.34
Level change after MPL interventions (β_2_)	−1.12	3.38	−0.33	0.11
Trend change after MPL interventions (β_3_)	−0.88	0.33	−2.66	0.01
PRA (%) (DW =1.97)				
Level before MPL interventions (β_0_)	16.23	1.84	8.82	0.00
Trend before MPL interventions (β_1_)	0.11	0.15	0.70	0.49
Level change after MPL interventions (β_2_)	4.95	2.27	2.18	0.038
Trend change after MPL interventions (β_3_)	−0.45	0.22	−2.08	0.046
PRCA (%) (DW =2.08)				
Level before MPL interventions (β_0_)	29.83	3.44	8.67	0.00
Trend before MPL interventions (β_1_)	0.24	0.29	0.82	0.42
Level change after MPL interventions (β_2_)	−9.91	4.25	−2.33	0.03
Trend change after MPL interventions (β_3_)	−0.08	0.41	−0.18	0.86
ALoS (day) (DW =2.00)				
Level before MPL interventions (β_0_)	10.01	0.37	27.40	0.00
Trend before MPL interventions (β_1_)	−0.02	0.03	−0.58	0.56
Level change after MPL interventions (β2)	−1.79	0.45	−3.97	0.00
Trend change after MPL interventions (β3)	0.02	0.04	0.41	0.69

Residual analysis was used to examine the presence of serial autocorrelation and the four models had no autocorrelation. IAC, the intensity of antibiotic consumption; PRA, the proportion of receiving antibiotics; PRCA, the proportion of receiving combined antibiotics; ALoS, the average length of hospital stay; DW, Durbin-Watson statistic; MPL, Multifaceted pharmacist-led.

The intensity of antibiotic consumption (IAC) per month showed a slight ascending trend (coefficient = 0.35, *p* = 0.34) before the MPL intervention. After the intervention, the trend was descending (coefficient = −0.88, *p* = 0.01), and the difference was statistically significant. There were no significant differences before and after MPL intervention in the level of change in the IAC.

A declining secular trend (coefficient = −0.45, *p* = 0.05) and an increase in the level of change (coefficient = 4.95, *p* = 0.038) were found in the proportion of patients receiving antibiotics (PRA) per month after the MPL intervention. The PRA had a brief increase in level change, but showed a significant descent in the secular trend.

The proportion of patients receiving combined antibiotics (PRCA) per month presented a noteworthy decrease in the level of change (coefficient = −9.91, *p* = 0.03) after the MPL intervention. Similarly, the decreasing change in the level of ALoS was statistically significant (coefficient = −1.79, *p* = 0.00). The MPL interventions slightly reduced the trend of PRCA (coefficient = −0.08, *p* = 0.86) and a slightly increased the trend of ALoS (coefficient =0.02, *p* = 0.69), but these effects were not statistically significant.

### Discussion

This study provides an evidence for the effect of MPL interventions on antibiotic use and length of hospital stay in a gastroenterology department in the Xinjiang province of China. The segmented regression analysis of a 3-year ITS indicated that the MPL intervention was associated with a significant decline in the secular trend of IAC and PRA, as well as an immediate drop in the level change of PRCA and ALoS. The advantage of the segmented regression analysis of ITS is the ability to control for prior trends in the outcome and study the dynamics of change (Wagner et al., 2002; Taljaard et al., 2014), which fully explained the instantaneous influences and potential long-term effects of the interventions implemented by the clinical pharmacist.

In this study, four indicators were used to measure the effectiveness of MPL interventions, which will be discussed in more detail.

Firstly, reducing the consumption of antimicrobials. Pharmacists’ interventions in ASPs have been attested to be associated with a reduction in antibiotic consumption (Magedanz et al., 2012; Cappelletty and Jacobs, 2013; Brink et al., 2016). In this study, the changes of IAC in the slope of the time series after the MPL intervention were 0.88 defined daily doses/100 bed-days per month. In an Italian GW, intervention by a multidisciplinary ASP team reduced the consumption of antibiotics and improved the clinical outcomes (Bedini et al., 2016). Although the consumption of antibiotics in the GW is not particularly high compared to other departments, bacterial resistance is still a problem (Fernandez et al., 2016; Piano et al., 2019). Therefore, this MPL intervention of ASPs in the GW was an important supplement to existing research.

Secondly, we observed the changes in the percentage of patients receiving antibiotics. The PRA and PRCA indices, which are used by the Chinese government to evaluate the effects of clinical application management of antibacterial therapeutics (National Health Commission of the People’s Republic of China, 2015b), were also used by scholars to analyze prescribing practice (Yang et al., 2014; Liu et al., 2019). In this study, the PRA and PRCA presented a decreasing trend and reduced levels after intervention.

Thirdly, a declining level in the ALoS was observed. Some studies found that the ALOS was shorted after the intervention of a clinical pharmacist (Shen et al., 2011), while some studies indicated that the intervention had no effect on the ALoS (Bedini et al., 2016; Wenzler et al., 2017), which may be related to differences in the study design and analytical methods.

Changes in prescription behavior require multifaceted interventions. The decision to prescribe an antibiotic is influenced by many factors, and professionals play an important role (van der Meer and Grol, 2007). Studies found that physicians feared treatment failure and, therefore, routinely prescribed broad-spectrum antibiotics for surgical prophylaxis.as well as frequently switched and used combinations (Livorsi et al., 2015; Gebretekle et al., 2018). Moreover, these habits were not easily changed (Furst et al., 2015), whereby multifaceted interventions with activities at a variety of levels were the most successful in reducing antibiotic prescriptions (Arnold and Straus, 2005; van der Meer and Grol, 2007). Pharmacist-driven ASPs could positively influence doctors’ prescribing behavior and perceptions of antibiotic use (Ansari et al., 2003; Shen et al., 2011; Magedanz et al., 2012). This MPL intervention in the GW reaffirmed the need and urgency to expand the involvement of clinical pharmacists in ASPs.

Our research focused on ASPs of digestive diseases that have not received widespread attention. Based on a comparison of 3 years post-intervention to the pre-intervention phase, we found that the MPL intervention reduced the use of antimicrobials and shortened the length of hospital stay for digestive diseases, suggesting that antimicrobial management in non-priority departments also needs to be strengthened. Furthermore, the segmented regression of the ITS analysis techniques could estimate the influence of intervention more accurately than a simple pre-post comparison (Sun, 2013).

Nevertheless, this study also has some limitations. Firstly, our study used retrospective data from an information system, which did not include the patients’ condition and details of the pharmacists’ intervention, such as medication appropriateness. Consequently, we were unable to evaluate the specific situation of guidance given by the clinical pharmacists to the physician, which will be a goal of our further research. Secondly, this study did not include a simultaneous control group, since there was only one gastroenterology department treating the same diseases in this hospital, and it was difficult to control the consistency of the pharmacists’ intervention measures in the gastroenterology departments of different hospitals. Therefore, we only made a comparative before and after analysis.

## Conclusions

This study provides a segmented regression analysis of the effects of involving a clinical pharmacist in reducing the use of antimicrobial agents and length of hospital stay in a gastroenterology department of a Chinese hospital. After the MPL intervention, the utilization of antibiotics was reduced, and the average length of hospital stay was shortened. Our data indicate that departments that are not a priority for antimicrobial administration also need attention. It is very important for local or regional policymakers to practice multiple interventions by clinical pharmacists in ASPs to improve the health care system.

## Data Availability Statement

All datasets generated for this study are included in the article/supplementary material.

## Ethics Statement

The study was approved by the Ethics Committee of Tongji Medical College, Huazhong University of Science and Technology, Wuhan, Hubei, China. Because the study used anonymous, aggregated, and retrospective data, the ethics committee waived the need for written informed consent provided by participants.

## Author Contributions

YD, JL, XZ, XinW, XP, and XiaW contributed with the design and conception of the study. QY, YL, WH, and XueW made significant contributions to the data interpretation. XZ, JL and XinW contributed to the revision of the manuscript. All authors read and approved the final manuscript as submitted.

## Funding

This study was supported by the National Natural Science Foundation of China, “Theorising Pharmacist-Patient Communication Model and Mechanism Based on King’s Theory of Goal Attainment” (Grant Number G040602, 2017).

## Conflict of Interest

The authors declare that the research was conducted in the absence of any commercial or financial relationships that could be construed as a potential conflict of interest.
